# ASO Author Reflections: Can Patient Selection for Cytoreductive Surgery and Hyperthermic Intraperitoneal Chemotherapy be Improved?

**DOI:** 10.1245/s10434-019-07664-6

**Published:** 2019-09-13

**Authors:** Wilhelm Graf, Helgi Birgisson

**Affiliations:** Department of Surgical Sciences, Akademiska Sjukhuset, Uppsala University, Uppsala, Sweden

## Past

Despite improving median and 5-year survival in patients with peritoneal surface malignancy originating from the large bowel, a substantial proportion experience rapid disease progression after seemingly radical cytoreductive surgery (CRS) and hyperthermic intraperitoneal chemotherapy (HIPEC). Some of these patients have low Peritoneal Cancer Index (PCI). Various more or less fruitful attempts have been undertaken to predict outcome but, to date, reliable measures are insufficient. Among useful predictive tools are extent of peritoneal dissemination, signet cell differentiation, and colorectal peritoneal (COREP) score.^1^^–^3

## Present

Our study identified BRAF mutation as a potential predictive tool in one-tenth of patients scheduled for CRS and HIPEC.^4^ In fact, no patients with BRAF mutation experienced long-term survival. Mutated KRAS did not influence survival in patients undergoing CRS and HIPEC. The predictive importance of BRAF mutation also remained in multivariate analysis adjusted for PCI, CCS, and signet cell differentiation.

## Future

The present results need to be verified in an independent and larger patient sample. If BRAF mutation can be confirmed to have this poor prognosis after CRS and HIPEC, BRAF-mutated subjects may need alternative therapeutic strategies, such as systemic chemotherapy or targeted therapies with BRAF inhibitors, combined with CRS and HIPEC. The efforts to select the most suitable group for CRS and HIPEC should be continued, and even increased, in view of the results of a recent controlled trial^5^ where unselected patients were included.
